# The Effects of Long-Term, Low- and High-Dose Beta-Carotene Treatment in Zucker Diabetic Fatty Rats: The Role of HO-1

**DOI:** 10.3390/ijms19041132

**Published:** 2018-04-10

**Authors:** Evelin Csepanyi, Attila Czompa, Peter Szabados-Furjesi, Istvan Lekli, Jozsef Balla, Gyorgy Balla, Arpad Tosaki, Istvan Bak

**Affiliations:** 1 Department of Bioanalytical Chemistry, Faculty of Pharmacy, University of Debrecen, 4032 Debrecen, Hungary; csepanyi.evelin@pharm.unideb.hu (E.C.); szabados-furjesi.peter@pharm.unideb.hu (P.S.-F.); 2 Department of Pharmacology, Faculty of Pharmacy, University of Debrecen, 4032 Debrecen, Hungary; czompa.attila@pharm.unideb.hu (A.C.); lekli.istvan@pharm.unideb.hu (I.L.); tosaki.arpad@pharm.unideb.hu (A.T.); 3 Hemostasis, Thrombosis and Vascular Biology Research Group, Hungarian of Academy of Sciences, 4032 Debrecen, Hungary; balla.jozsef@med.unideb.hu (J.B.); balla@med.unideb.hu (G.B.)

**Keywords:** β-carotene, diabetes, heart, ischemia/reperfusion, heme-oxygenase-1

## Abstract

Nowadays, there is a growing interest in compounds derived from plants as potential raw materials for drug development. One of the most studied compounds is beta-carotene (BC). Several clinical studies can be found investigating the cardiovascular effects of BC, however, all these results are controversial. There is an increasing body of evidence showing that besides the well-known antioxidant properties, under strong oxidative circumstances, BC could become prooxidant as well. In this study, we investigated the effects of long-term, low- and high-dose BC treatment in ischemic/reperfused (ISA/REP) hearts isolated from Zucker diabetic fatty (ZDF) rats. The animals were treated with various daily doses of BC for 4 weeks and then hearts were isolated and subjected to 30 min of global ischemia (ISA) followed by 120 min of reperfusion (REP). Blood glucose levels were measured before, after two weeks, and at the end of the treatment. In isolated hearts, the myocardial function was registered. At the end of the reperfusion period, the infarct size (IS) and heme oxygenase-1 (HO-1) expression were measured. The results showed that a low dose of BC treatment significantly improved postischemic recovery, which was reflected in a decreased IS. Interestingly, when BC was applied at high concentrations, the observed protective effects were lost. Although BC treatment increased HO-1 expression, we did not observe a better heart function and/or decreased IS in the high-dose-treated group. Glucose tolerance tests showed a concentration-independent decrease in blood glucose levels. Our results suggest that long-term, low-dose BC treatment could be effective in the treatment of type-2-diabetes and related cardiovascular diseases.

## 1. Introduction

A variety of materials of plant origin have been used for the treatment of different diseases. Nowadays, there is a growing interest in compounds derived from plants for the prevention and/or treatment of various disorders. Many plants contain flavonoids, anthocyanidins, proanthocyanidines, and carotenoids, which are all well-documented antioxidants and help in other biological activities [1,2,3,4,5,6].

In the past decades, there has been a significant increase in the number of patients diagnosed with type-2-diabetes (T2D), which is a multifactorial disease and one of the major risk factors for the development of cardiovascular diseases [7,8,9]. Besides the application of standard pharmaceutical drugs, the first step in the treatment of T2D is changing one’s lifestyle, including diet. Several research groups have studied the effects of different antioxidants in the management of T2D and related complications, hence, it has been theorized that the pathogenesis of T2D involves strong oxidative stress with the increased formation of free radicals and decreased antioxidant status of tissues [10]. In previous experiments, we showed that resveratrol could help to prevent, or at least delay, the occurrence and complications caused by myocardial ISA/REP-induced damage in connection with the insulin-resistant state in rats [11]. Chen K. et al. found that berberine, an isoquinoline derivative alkaloid, reduces ISA/REP-induced cardiovascular damage in diabetic rats [12]. Furthermore, Zhang H. et al. observed that berberine has a beneficial effect on blood glucose levels [13]. Moreover, it had been shown that vitamin E and coenzyme Q10 could be effective in the prevention and treatment of T2D, while vitamin C could play a role only in the acute treatment [14]. Another well-known antioxidant molecule is BC. Although BC has well-documented antioxidant properties and cardiovascular effects, many studies show that it is ineffective in the prevention or treatment of T2D [10,14,15]. Furthermore, there is an increasing amount of evidence showing the prooxidant properties of BC under different pathophysiological conditions [16,17,18]. Hence, during the increased oxidative circumstances, harmful derivatives are formed which can further increase cellular and tissue damage [19,20,21]. Earlier, we investigated the effects of low- and high-dose BC in ischemic/reperfused non-diabetic rat myocardium [18]. In those experiments we found that low-dose (LD) BC significantly improved postischemic cardiac function, reduced IS, and increased tissue antioxidant capacity, while these effects were abolished when BC was administered at high concentrations. Furthermore, high-dose (HD) BC treatment resulted in a significant elevation in HO-1 expression. Moreover, an increasing body of evidence shows that HO-1 may have an important role in diabetes and glucose metabolism [22,23,24,25]. Although HO-1 is considered a cytoprotective enzyme, some studies show that it may have prooxidant properties [26,27] and hence, could exacerbate diabetes-related increased oxidative stress.

Depending on the circumstances and the concentration of BC, the agent could function as an antioxidant or a prooxidant [19]. The observations that the agent, applied at high concentrations, causes the loss of the cardioprotective effects in hearts subjected to ISA/REP suggests that under those conditions, the BC could become prooxidant [18]. Hence, diabetes is considered as having a strong oxidative condition, and we hypothesized that along with ISA/REP, it can further enhance the prooxidant activity of BC. Because the data on the effects of BC in the diabetic ischemic/reperfused hearts are limited in the literature, in the present study, we investigated the effects of long-term BC treatment in diabetic rats in order to get further information about the pathological mechanisms of cardiovascular damages associated with T2D in the ischemic myocardium. Furthermore, because HO-1 is induced in response to various interventions that cause oxidative stress, we studied the expression of the enzyme in response to ISA/REP. Therefore, the present study was undertaken to determine the effects of 4 weeks of treatment with low- and high-dose BC on postischemic cardiac function, IS, and HO-1 expression after 30 min of ISA followed by 120 min of REP in Zucker obese rat hearts to study the effects of BC in T2D related increased oxidative circumstances. In addition, we investigated the effect of BC on the blood glucose level.

## 2. Results

### 2.1. The Effect of BC Treatment on the Cardiac Function in Isolated Hearts Subjected to ISA/REP

**Figure 1:**
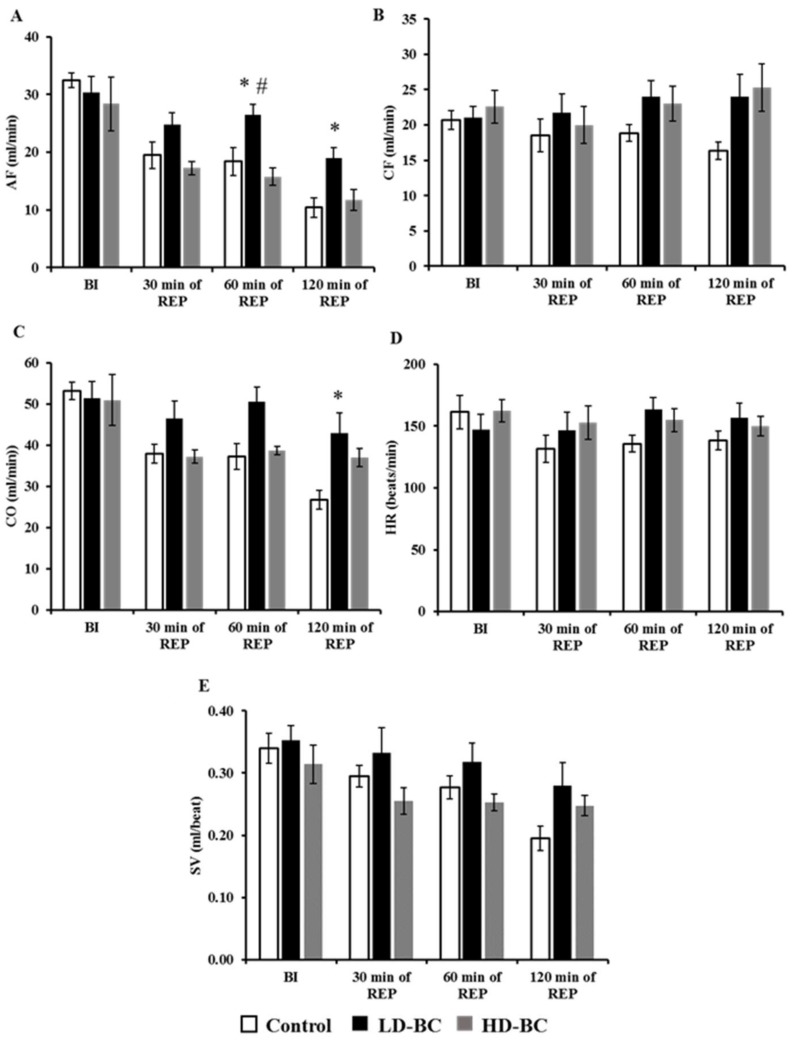
The beta-carotene (BC) effects on cardiac function in isolated working hearts. Hearts were isolated from rats (*n* = 8 per group) received hydroxyethyl cellulose-water (1:4) as a vehicle control (open bars); BC 30 mg/kg/day (black bars); or BC 150 mg/kg/day (gray bars) for 4-week time periods. Isolated hearts were subjected to 30 min of global ISA followed by 120 min of REP in an isolated “working-heart” apparatus. Results are shown as the mean ± SEM of AF (**A**); CF (**B**); CO (**C**); HR (**D**), and SV (**E**). * *p* < 0.05 in comparison with the magnitude of each cardiac function measured in each test group receiving BC, relative to the hearts of vehicle-treated control animals. ^#^ *p* < 0.05 for comparison of the magnitude of cardiac function measured in LD-BC relative to the hearts from HD-BC treated animals. BC: beta-carotene, BI: before ISA, ISA: ischemia, REP: reperfusion, AF: aortic flow, CF: coronary flow, CO: cardiac output, HR: heart rate, SV: stroke volume.

Figure 1 shows the effect of 0, 30, or 150 mg/kg/day BC on the cardiac function of hearts subjected to 30 min of global ISA and 120 min of REP. No BC-mediated effects on the aortic flow (AF) were observed in the hearts before ISA/REP. When compared to the hearts from the vehicle-treated control animals, hearts from the LD-BC-treated rats subjected to 30 min of ISA followed by 120 min of REP exhibited a significant increase in AF (* *p* < 0.05); but interestingly, not from those fed with HD-BC (Figure 1A). Furthermore, LD-BC treatment resulted in a substantial increase in AF after 60 min of REP compared to the hearts isolated from animals treated with HD-BC (^#^ *p* < 0.05) (Figure 1A). No significant BC dosage effects on the coronary flow were observed in the hearts either before ISA or after 30 min of ISA followed by 120 min of REP (Figure 1B). However, BC treatment resulted in a moderate increase in coronary flow values after 120 min of REP relative to the organs from non-BC-treated controls, independent from the dose; but this elevation did not reach a significant level (Figure 1B). Likewise, for cardiac output, we did not observed differences between the control and test groups before the induction of ISA and after 30 min and 60 min of REP, whereas a significant increase in the cardiac output of the hearts, relative to the organs from non-BC-treated controls were observed in those that sustained reperfusion periods of 120 min (* *p* < 0.05) from rats receiving 30 mg/kg/day BC, but not 150 mg/kg/day of the agent (Figure 1C). 

The treatment of animals with BC did not result in any changes in heart rate (Figure 1D). The evaluation of the effects of BC on the heart stroke volume revealed no significant BC effect on this variable before or after ISA/REP. It was further noted that the stroke volume values in hearts receiving 30 and 60 min of REP from animals treated with 150 mg/kg/day of BC were reduced, however, it was not to a significant level relative to those from rats receiving LD-BC (Figure 1E).

### 2.2. The BC Dose Effects on ISA/REP-Induced IS

**Figure 2:**
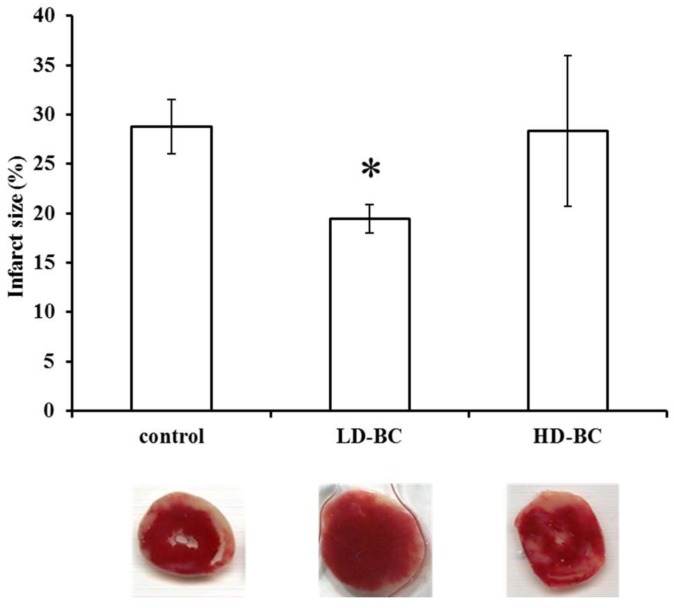
The effect of BC on infarcted zone magnitude. IS was measured in hearts (*n* = 4) following 120 min of reperfused (REP) by perfusion with triphenyl-tetrazolium-chloride (TTC) solution, followed by macroscopic analysis of transverse sections of each heart. The average size (%) of the infarcted area ±SEM are shown for each group. * *p* < 0.05 in comparison with the values for hearts from vehicle-treated control animals. Representative pictures are also shown under the bars.

The effect of BC treatment on the extent of infarct size after ISA/REP is shown in Figure 2. Macroscopic analysis of triphenyl-tetrazolium-chloride (TTC) solution-perfused heart sections revealed that LD-BC treatment correlated with a significant reduction in the extent of infarcted myocardium relative to the control values (* *p* < 0.05). This protective effect is abolished in hearts taken from animals treated with HD-BC.

### 2.3. The BC Effects on HO-1 Protein Expression

**Figure 3:**
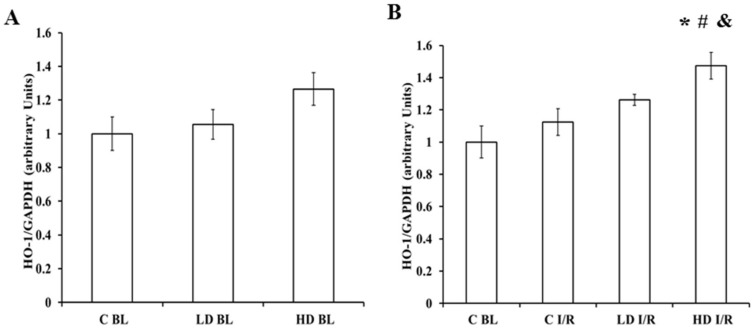
The Western blot analysis for cardiac expression of the HO-1 protein. Expression of the HO-1 protein in rat myocardiums was measured in the homogenized cardiac tissue samples from vehicle- or BC-treated hearts, with (**B**) or without (**A**) ischemic/reperfused (ISA/REP) injury. The signal intensity of the resulting bands corresponding to the HO-1 protein was measured using the Scion for the Windows Densitometry Image program. The tissue content of each protein is shown as a ratio of arbitrary units for the HO-1 protein to GAPDH signal. Data are expressed as the mean ± SEM of the 6 different blots. * *p* < 0.05 for the comparison of the average levels of HO-1 in the ventricular myocardium of BC-treated animals versus non-ischemic control (C-BL) hearts. ^#^ *p* < 0.05 for the comparison of the average levels of HO-1 in the ventricular myocardium from BC-treated animals subjected to ISA/REP versus ISA/REP-injured control (C-I/R) hearts. ^&^ *p* < 0.05 for the comparison of the average levels of HO-1 in the ventricular myocardium from high dose beta-carotene treated (HD-BC) animals subjected to ISA/REP (HD-I/R) versus LD-BC treated and ISA/REP-injured (LD-I/R) hearts.

The myocardial tissue levels of HO-1 were determined by Western blot analysis (Figure 3). Hearts excised from animals were either vehicle-treated (BL-baseline) (Figure 3A) or ISA/REP-injured (I/R) (Figure 3B). The results show that in control hearts, which were not subjected to ISA/REP (C-BL), the HO-1 expression was not significantly different from non-injured hearts removed from rats receiving either low- or high-dose BC (Figure 3A). Furthermore, the production of the HO-1 protein in ISA/REP-injured hearts from rats fed with high-dose BC (HD-I/R) was significantly higher compared to the non-treated intact (C-BL) group (* *p* < 0.05) (Figure 3B). Moreover, the HO-1 expression in hearts removed from vehicle-treated animals and subjected to I/R (C-I/R) was not significantly different from hearts excised from rats receiving LD-BC and subjected to I/R (LD-I/R); however, hearts from high-dose treated animals expressed significantly elevated levels of HO-1 protein relative to corresponding levels measured in vehicle-treated controls (C-I/R) (^#^ *p* < 0.05) or LD-BC-treated animals (LD-I/R) (^&^ *p* < 0.05) (Figure 3B).

### 2.4. The Effects of BC on the Blood Glucose Level

**Figure 4:**
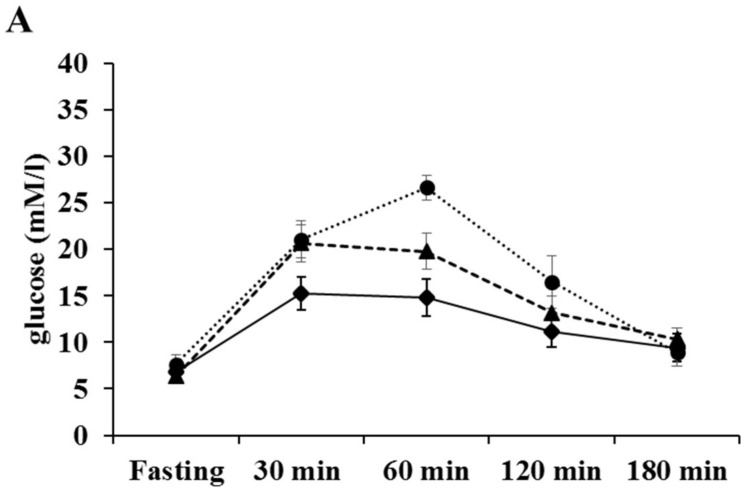

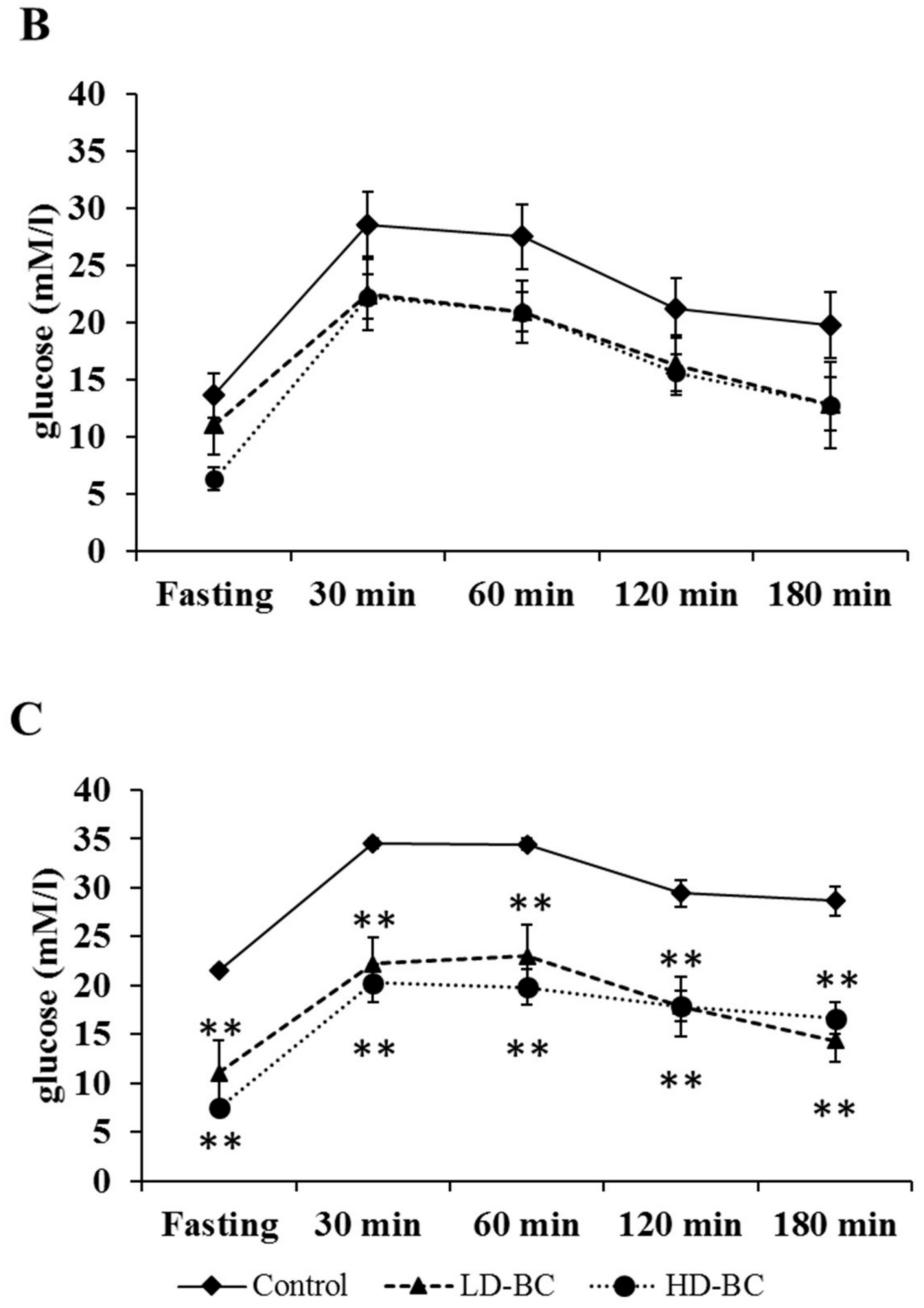
The effect of BC treatment on blood glucose level before (**A**), at halftime (**B**) and at the end (**C**) of the treatment period. The animals (*n* = 10 animals per group), fed daily by gavage for 4 weeks with a mucin-water vehicle or BC were evaluated for their fasting serum glucose content immediately prior to oral administration of 3 g of glucose per kg of their body weight. The results are provided as an average serum glucose content, in mmol/L of peripheral blood, ±SEM. ** *p* < 0.005 control versus BC treated groups.

The results of the oral glucose tolerance test are shown in Figure 4. We did not observe differences before (Figure 4A) and at halftime (Figure 4B) of the treatment period among the different groups of animals, while at the end of treatment BC resulted in a significant decrease (** *p* < 0.005) in blood glucose level which was independent from the applied dose of BC (Figure 4C).

## 3. Discussion

The number of diabetic patients is increasing continuously worldwide [7]. Obesity and overweight related to the lack of physical activity are considered among the major factors for the development of diabetes [28]. Several strategies, including diets, weight loss, and physical exercise are suggested to reduce the risk of developing the disease [28]. Diabetes and the increased oxidative stress associated with it are major risk factors for the development of cardiovascular disorders [8,9]. Hence, because oxidative stress is mainly caused by free radicals and reactive oxygen and nitrogen species, it is reasonable to assume that different antioxidants such as vitamin E and C or β-carotene could prevent or at least delay the development and/or progression of diabetes-related cardiovascular diseases [29,30]. In this study, we investigated the effects of long-term BC treatment in the hearts isolated from ZDF rats. Although BC is considered a good antioxidant compound, there is an increasing amount of evidence suggesting that it has prooxidant effects under heavy oxidative stress. In earlier studies, we investigated the effects of the agent under ISA/REP-induced increased oxidative circumstances [18]. In that study, we observed that the cardioprotective effects of BC were lost after ISA/REP when the agent was administered at high concentrations. If diabetes further enhances the ISA/REP-induced oxidative stress, then the effects of BC should be similar or even worse compared to non-diabetic ISA/REP-injured hearts. The results are in good correlation with our earlier observations since we found that LD treatment resulted in increased aortic flow after 60 min and 120 min and increased cardiac output after 120 min of REP compared to drug-free control hearts. Note that these positive effects were abolished in the case of HD treatment (Figure 1). Furthermore, after 60 min of REP, the treatment with LD-BC resulted in significantly higher aortic flow values compared to the HD-BC treated hearts supporting the diabetes enhanced oxidative effort of ISA/REP. This hypothesis is further supported by the outcome of infarcted area measurements. Thus, the administration of LD-BC to the rats resulted in decreased infarct size compared to hearts isolated from vehicle-treated animals. This observation is indirectly supported, however, by the results of Da Rocha et al. who investigated the effects of long-term vitamin A treatment in rat hearts [31]. They found that after 28 days of treatment with retinyl-palmitate, the highest dose increased lipid and protein oxidation. Furthermore, they observed decreased catalase activity, decreased SOD/CAT ratio, and reduced mitochondrial respiratory chain complex activities. Hence, BC, one of the major sources of vitamin A, is converted to retinal, and then to retinol by the central or eccentric cleavage in vertebrates [32,33]. Thus, it is reasonable to assume that it will result in similar effects which support our findings on IS. The consequences of the decreased catalase activity include the accumulation of H_2_O_2_ in the tissue and in the presence of transition metals, especially Fe^2+^; it could result in the generation of hydroxyl radicals in excess and induce cellular and tissue damage which is shown in the loss of the infarct size limitation capacity of HD-BC (Figure 2). This explanation is strengthened by the measured increased HO-1 expression in hearts isolated from HD-BC-treated animals subjected to ISA/REP (Figure 3B) because Fe^2+^ is also produced by HO-1 activity. This is further supported by some studies that show the prooxidant role of the HO system in diabetes [26,27]. The authors of these studies showed that short-term diabetes-induced HO-1 expression and activity which was associated with increased 8-OH-dG (a marker of the oxidative stress) levels, and the observed effects could be reversed by the application of SnPPIX, a well-known HO inhibitor. Moreover, the increased HO activity is also associated with the accumulation of Fe^2+^ in the cardiac tissue [27]. Normally, increased activity of HO-1 reduces oxidative stress and protects tissues from oxidative insult. In our present study, ISA/REP-induced injury to cardiac tissue along with the high BC dosage strongly activates HO-1 (Figure 3B), which could result in elevated levels of Fe^2+^. Hence, it was demonstrated that Fe^2+^ enhances the formation of different BC cleavage products [34], influences the elevated HO-1 in the presence of high BC levels, potentially overwhelms the cytoprotective effects of HO-1 and increases the oxidative stress in cardiac tissue.

On the other hand, the increased HO-1 expression and activity could be protective in diabetes and related complications. Ndisang J.F. et al. showed that the strong induction of the enzyme improved glucose metabolism and reduced blood glucose level [22,24]. In other studies, the induction of HO-1, which was also associated with reduced blood glucose levels, ameliorated vascular dysfunction [25], and reduced hyperglycemia-induced renal injury [23] in diabetic rats. In our study we observed a dose-independent reduction in fasting blood glucose levels; however, this could not be the result of only the increased HO-1 expression. Although we found a slight increase in HO-1 expression at the end of the treatment period (Figure 3A), it did not reach a significant level; hence, the blood glucose lowering effect could mainly be the result of BC treatment. This observation is in good correlation with the results of different research groups. Furusho T. et al. fed rats with a 0.1% BC containing diet for 28 days [35] while Ma Q.Y. et al. treated mice with purified BC (100 mg/bwkg, daily) for 10 days, which was extracted from *Spirulina platensis* [36]. In both cases, the BC treatment resulted in a significant decrease in blood glucose level.

It has to be mentioned that the data presented here do not allow a comprehensive mechanistic analysis and the conclusions are rather speculative. The main limitation of the present study originates from the experimental model. Although a rat model was selected for the present study based on the well-known characteristics of rats and its widespread application in cardiovascular research, the experiments were carried out in the context of known limitations on the translational value of carotenoid research using rats. An important factor is the poor bioavailability of the carotenoids, including BC, in case of the absorption from the gut requiring the administration of BC at higher dosages than physiologically relevant. In the present study, even the lower dose of BC (150 mg/bwkg) administered to the animals is very high, hence a normal human diet may contain three orders of magnitude less daily BC [18]. Since this study was designed to obtain mostly qualitative information about the effects of low-, versus high-dose BC on the extent of ISA/REP-induced cardiac injuries, no attempt was made to determine exact rat-to-human dose equivalents. Accordingly, use of the findings of this study in the design of primary and/or secondary prevention strategies must be made carefully, taking into account experimental versus normal dietary or therapeutic dose ranges [18].

In conclusion, the main findings of the results show that increasing the concentration of BC will not provide an additional benefit in the diabetic ischemic/reperfused myocardium. Our results suggest that the level of Fe^2+^ produced by the increased HO-1 activity in response to ISA/REP injury may be a critical factor in the role of BC in ISA/REP-induced heart damage, which could act either as a protective antioxidant agent or behave as a prooxidant molecule and accelerate ISA/REP-related syndromes. This conclusion is speculative and will require further analysis. The ongoing characterization of the role of Fe^2+^ related to HO-1 activity is under investigation by our laboratory. Furthermore, our work demonstrated that BC possesses a hypoglycemic effect. The role of HO-1 in mediating the cardiovascular effects of BC and the mechanism behind the hypoglycemic action of the agent needs to be further elucidated.

## 4. Materials and Methods

The experiments were accomplished using adult male obese ZDF rats (Charles River Laboratories, Wilmington, MA, USA) with a body weight range of 350–400 g. The animals were fed with special rodent chow (Purina #5008) according to the supplier instructions in order to induce programmed and consistent development of T2D. All animals received humane care in compliance with the “Principles of Laboratory Animal Care” (formulated by the U.S. National Society for Medical Research, as described in U.S. National Institutes of Health, publication No. 86-23, revised 1996) and the “Guide for the Care and Use of Laboratory Animals”. The maintenance and treatment of animals used in the present study were additionally approved by the Institutional Animal Care and Use Committee of the University of Debrecen, Debrecen, Hungary (3/2012/DE MAB, 03/2012–03/2017). The rats were housed in wire-bottomed cages (three rats per cage) throughout the study and were maintained on a 12:12-h light-dark cycle and provided with laboratory rodent chow pellets and water ad libitum.

### 4.1. Animal Treatment

The rats used in the present study were segregated into 3 groups (*n* = 10/group) and treated by gavage with the following agents: hydroxyethyl cellulose-water (1:4) vehicle control (C), LD-BC (30 mg/kg/day), and HD-BC (150 mg/kg/day) suspended in hydroxyethyl cellulose-water, respectively. It has to be mention that the applied doses exceed the levels from any natural sources of BC. Although rodents (rats and mice) readily convert BC to vitamin A, the bioavailability of the agent is low in case of absorption through the gut, requiring administration of BC at higher doses than physiologically required. The experiments were designed to partly offset this limitation. All-trans-BC was obtained from Sigma-Aldrich Kft. (Budapest, Hungary). Table 1 shows the number of hearts used for the different measurements.

### 4.2. Isolated Working Heart Preparation

Following the 4-week treatment with the vehicle or BC, the animals were anesthetized with an intraperitoneal ketamine-xylazine injection (75/10 mg/kg), and intravenous heparin was given as an anticoagulant (1000 IU/kg). After thoracotomy, the hearts were excised and placed in ice-cold modified Krebs-Henseleit bicarbonate buffers. The isolated working rat heart model was used which has been previously described in detail [37]. Briefly, the aorta was cannulated and the Langendorff perfusion (100 cm of water, 10 kPa) initiated. During the Langendorff perfusion, the pulmonary vein was cannulated for the preparation for the conversion of the heart to the working heart mode which was achieved by stopping the Langendorff perfusion and starting left atrial perfusion (at a filling pressure of 17 cm of the buffer). Under these conditions, the perfusate was ejected spontaneously through the aortic cannula against a hydrostatic pressure of 100 cm of the perfusion buffer. The perfusion buffer consisted of a modified Krebs-Henseleit bicarbonate buffer (millimolar concentrations: NaCl 118, KCl 5.8, CaCl_2_ 1.8, NaHCO_3_ 25, NaH_2_PO_4_ 0.36, MgSO_4_ 1.2 and glucose 5.0).

### 4.3. Induction of ISA/REP and Measurement of Cardiac Functions

After 10 min of working perfusion, 30 min of global ischemia was initiated by clamping the pulmonary inflow and the aortic outflow. At the end of the ischemic period, 120 min of reperfusion was started by unclamping the inflow and outflow lines. The first 10 min of reperfusion was conducted in Langendorff mode to avoid the development of fatal ventricular arrhythmias as described by Reference [18]. The baseline parameters for each heart were registered following the 10 min of working perfusion before the induction of ischemia. To examine the recovery of the left ventricle, cardiac function was assessed after 30, 60, and 120 min of reperfusion during the entire experimental procedure, the heart rate was measured by a computer acquisition system (ADInstruments, PowerLab, Castle Hill, Australia). The aortic flow was measured with the help of a calibrated flow meter. The coronary flow was measured by the timed collection of the effluent dripping from the heart. The cardiac output was generated as a sum of aortic flow and coronary flow. The stroke volume was calculated as the quotient of cardiac output/heart rate [18].

### 4.4. The Determination of IS

Estimations of the IS were conducted using the triphenyl tetrazolium chloride (TTC) staining method. Briefly, following each 30 min ischemia/120 min reperfusion period, the hearts were perfused with a 50 mL 1% (*w*/*v*) solution of TTC in a phosphate buffer (pH 7.4) and the samples were stored at −70 °C for subsequent analysis. The frozen samples were sectioned, weighted, and blotted dry. The dried sections were scanned on an Epson J232D flat-bed scanner (Seiko Epson Corporation, Nagano, Japan). The infarcted area (white coloration) and the risk area (entire scanned section) were measured using planimetry software (Image J, National Institute of Health, Bethesda, MD, USA). Estimates of the infarcted zone magnitude were subsequently obtained by multiplying the infarcted areas by the weight of each slice. The resulting numbers represent the weight of the risk zone and the infarcted zone. The infarct size was expressed as a percentage of the weight of the infarcted tissue and the weight of risk zone (whole heart).

### 4.5. Western Blot Analysis

The content of the HO-1 protein in the myocardium was obtained by Western blot analysis. Approximately 300 mg of left ventricular myocardial tissue were homogenized on ice using a tissue homogenizer (IKA T10 basic ULTRA-TURRAX^®^, IKA^®^ Works, Inc., Wilmington, NC, USA) in an isolating buffer (25 mM Tris-HCl, 25 mM NaCl, 1 mM orthovanadate, 10 mM NaF, 10 mM pyrophosphate, 10 mM okadaic acid, 0.5 mM EDTA, 1 mM PMSF, and 1× protease inhibitor cocktail) and centrifuged at 2000 rpm at 4 °C for 10 min. The supernatants were transferred to fresh tubes and centrifuged at 10,000 rpm at 4 °C for 20 min, after which the resulting supernatant was used as the cytosolic fraction. The protein concentration was measured by ND-1000 Nano drop spectrophotometer with a BCA Protein Assay Kit (Thermo Scientific, Rockford, IL, USA). Thirty micrograms of protein in each sample were loaded in 10% polyacrylamide gel and resolved using SDS-PAGE electrophoresis and then transferred to a 0.45 µm pore size nitrocellulose membrane to concentrate the samples. After blocking the membranes with 7% nonfat dry milk in TBST, the membranes were incubated overnight with a primary antibody solution in 1% nonfat dry milk in TBST (GAPDH 1/40,000, the antibody was obtained from Cell Signaling Technology, Boston, MA; and HO-1 1/50 was ordered from Sigma-Aldrich Kft. Budapest, Hungary) at 4 °C. Then, the membranes were washed 3 times, each for 10 min, in TBST and incubated with a horseradish peroxidase-conjugated secondary antibody solution (Cell Signaling Technology, Danvers, MA, USA) containing 1% of nonfat dry milk in TBST, for two hours at room temperature. The membranes were treated with a Western blot Enhanced Chemiluminescent HRP substrate (Millipore, Billerica, MA, USA) to visualize the bands. After the Enhanced Chemiluminescent treatment, the membranes were exposed on X-ray films (Agfa, Mortsel, Belgium). The films were then digitalized by a flat-bed scanner (Epson J232D, Seiko Epson Corporation, Nagano, Japan) and analyzed using the ImageJ program and normalized the HO-1 band intensities to GAPDH.

### 4.6. Oral Glucose Tolerance Test (OGTT)

The blood glucose levels were measured before, at halftime, and at the end of the treatment period by a commercially available glucometer (Accu-chek Active, Roche Diabetes Care GmbH, Mannheim, Germany). The OGTT was performed as follows: after 12 h of fasting, the glucose levels were measured by sampling from the tail vein. Then, the rats were gavaged with 3 g/bwkg of glucose solution. The blood glucose levels were measured at the 30, 60, 120, and 180 min points after the administration of glucose.

### 4.7. Statistical Analysis

Statistical analyses were performed using the GraphPad Prism 5 software (GraphPad Software Inc., La Jolla, CA, USA). The data are expressed as the mean ± SEM. A one-way ANOVA followed by Newman-Keuls multiple comparison test was carried out for the data analysis. Any differences were considered significant at values of *p* < 0.05.

## Figures and Tables

**Table 1:** The number of hearts used in the different experiments.

	Total Number of Type II Diabetic Rats: 30		
Experimental procedure	Vehicle control	Low dose BC treated	High dose BC treated
	(C)	(LD-BC)	(HD-BC)
	10	10	10
Oral Glucose Tolerance Test (OGTT)	10	10	10
Ischemia/Reperfusion (ISA/REP)	8	8	8
Cardiac function measurement	8	8	8
Infarct Size (IS) determination	4	4	4
Western Blot analysis	5	5	5
